# The cGAS-STING pathway in cardiovascular diseases: from basic research to clinical perspectives

**DOI:** 10.1186/s13578-024-01242-4

**Published:** 2024-05-08

**Authors:** Cheng An, Zhen Li, Yao Chen, Shaojun Huang, Fan Yang, Ying Hu, Tao Xu, Chengxin Zhang, Shenglin Ge

**Affiliations:** 1 Department of Cardiovascular Surgery, The First Affiliated Hospital of Anhui Medical University, No.218 Jixi Road, Hefei, 230032 Anhui China; 2 Department of Orthopedics, The First Affiliated Hospital of Anhui Medical University, Hefei, Anhui China; 3 Department of Ophthalmology, The First Affiliated Hospital of Anhui Medical University, Hefei, Anhui China; 4 State Key Laboratory of Pharmaceutical Biotechnology, School of Life Sciences, Nanjing University, Nanjing, Jiangsu China; 5 Inflammation and Immune Mediated Diseases Laboratory of Anhui Province, Anhui Institute of Innovative Drugs, School of Pharmacy, Anhui Medical University, Hefei, Anhui China

**Keywords:** Cardiovascular diseases, cGAS-STING pathway, Inflammatory response, Risk factors, Inhibitors

## Abstract

The cyclic guanosine monophosphate (GMP)-adenosine monophosphate (AMP) synthase-stimulator of interferon genes (cGAS-STING) signaling pathway, an important component of the innate immune system, is involved in the development of several diseases. Ectopic DNA-induced inflammatory responses are involved in several pathological processes. Repeated damage to tissues and metabolic organelles releases a large number of damage-associated molecular patterns (mitochondrial DNA, nuclear DNA, and exogenous DNA). The DNA fragments released into the cytoplasm are sensed by the sensor cGAS to initiate immune responses through the bridging protein STING. Many recent studies have revealed a regulatory role of the cGAS-STING signaling pathway in cardiovascular diseases (CVDs) such as myocardial infarction, heart failure, atherosclerosis, and aortic dissection/aneurysm. Furthermore, increasing evidence suggests that inhibiting the cGAS-STING signaling pathway can significantly inhibit myocardial hypertrophy and inflammatory cell infiltration. Therefore, this review is intended to identify risk factors for activating the cGAS-STING pathway to reduce risks and to simultaneously further elucidate the biological function of this pathway in the cardiovascular field, as well as its potential as a therapeutic target.

## Introduction

Cardiovascular diseases (CVDs) are the leading cause of death worldwide and the number one cause of death in the United States [1]. The 2020 American Heart Association statistics show that approximately 19.05 million people died from CVDs worldwide, which was an increase of 18.7% from 2010 [2]. Previous surveys have shown that with the progress of industrialization and dietary changes, 37.4% of men and 35.9% of women over the age of 20 years in the United States have some form of CVDs, and men account for 50.6% of deaths from CVDs [1]. Innate and adaptive immunity play an important roles in CVDs, leading to inflammatory infiltration, abnormalities including apoptotic cells and autoantigen enhancement, and ultimately organ and functional damage [3, 4]. Notably, exogenous, host-damaged, and ectopic DNA enhances autoimmunity, leading to an enhanced inflammatory response and further exacerbation of cardiovascular injury [5, 6]. Therefore, finding a DNA sensor that recognizes DNA to intervene and simultaneously prevent risk factors for DNA damage are necessary.

Cyclic guanosine monophosphate-adenosine monophosphate synthase (cGAS), a cytoplasmic DNA sensor, is activated upon detection and binding of double-stranded DNA (dsDNA), thus catalyzing the synthesis of 2′3′-cGAMP. As a secondary messenger, 2′3’-cGAMP activates the stimulator of interferon genes (STING) and the transcription factor type-I interferon regulatory factor 3 (IRF3), which induces strong innate immunity [7]. cGAS is involved in important biological processes such as macular degeneration, cellular senescence, and myocardial infarction (MI), heart failure(HF), and cardiac hypertrophy [8–10]. The regulatory role of this signaling pathway in CVDs has attracted widespread attention. For example, Yan et al. [10] found that diabetic cardiomyopathy triggers cellular pyroptosis and activates the cGAS-STING signaling pathway, which promotes the production of type I interferon (IFN-I) and nucleotide-binding oligomerization domain-like receptor pyrin domain containing 3 (NLRP3) inflammasome. However, inhibition of this signaling pathway prevents the secretion of IFN-I and other proinflammatory cytokines [11]. In addition, studies have confirmed the success of the small-molecule inhibitor evolocumab, a proprotein convertase subtilisin/kexin type 9 (PSCK9) inhibitor, in treating hyperlipidemia in patients with CVD in phase IV trials (Clinical Trials.gov Identifier: NCT02867813 and NCT03080935), suggesting that targeted inhibition of an intracellular signaling pathway can treat CVDs [12]. Therefore, targeted small-molecule inhibitors designed against cGAS-STING signaling pathway may be a new strategy for the treatment of CVDs.

In this review, we describe the mechanism of the cGAS-STING signaling pathway in detail and highlight its role in different CVDs. Furthermore, the risk factors for the activation of cGAS-STING and the most clinically valuable specific small-molecule inhibitors developed in the cardiovascular field are reviewed.

## Overview of the cGAS-STING pathway

**Fig. 1:**
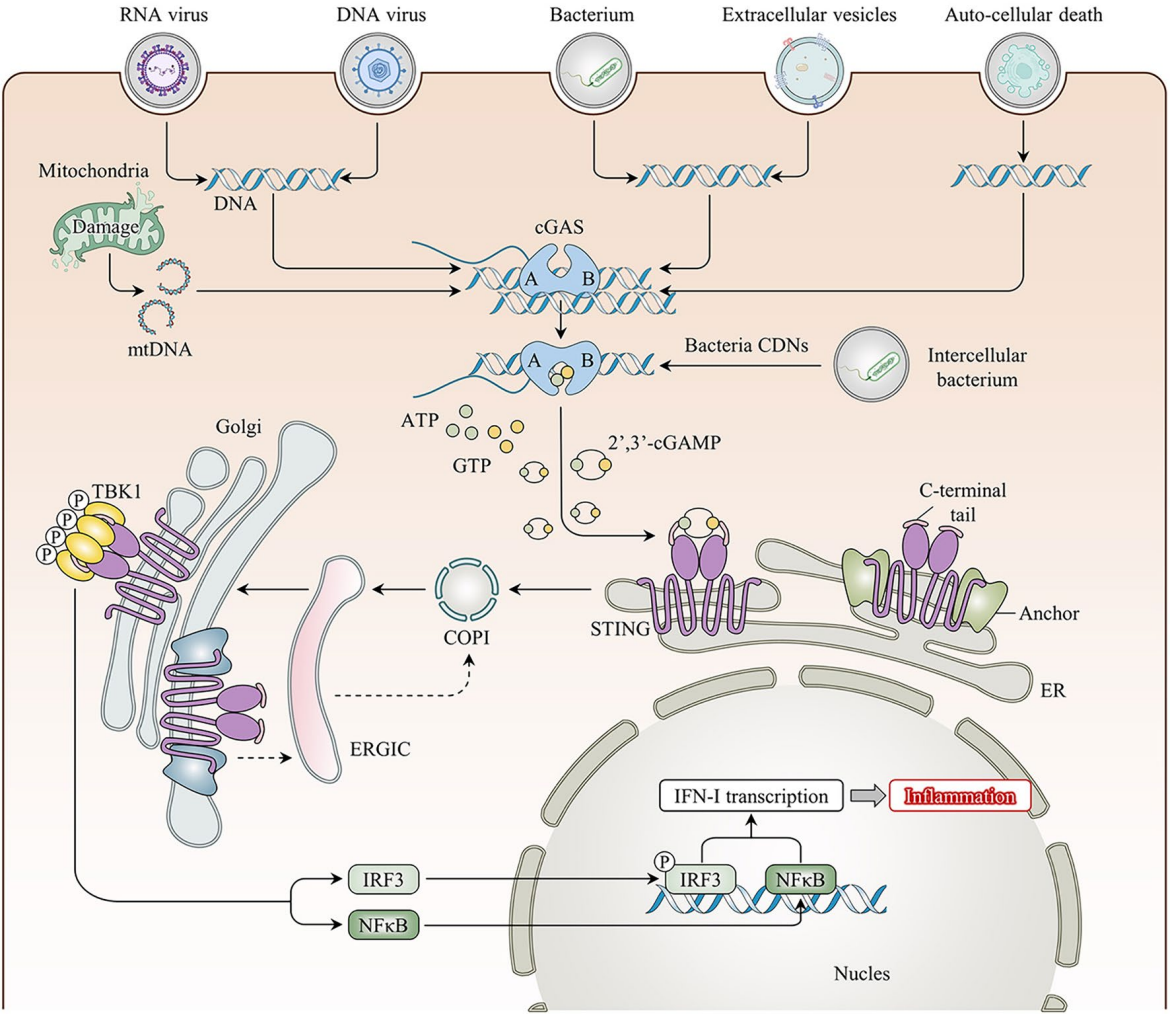
Overview of the cGAS-STING pathway. Exogenous (viral, bacterial) or endogenous (carried by extracellular vesicles, released by auto-cell death, intracellular mitochondrial damage) DNA binds to free cGAS in the cytoplasm and activates its biological dynamic, catalyzing the synthesis of 2′3′-cGAMP from ATP and GTP. Then, cGAMP binds to stimulator of interferon gene (STING) on the endoplasmic reticulum (ER) membrane resulting in a conformational change to activate its function. Activated STING transports to the Golgi apparatus through the coatomer protein complex I (COP I) and the ER–Golgi intermediate compartment (ERGIC), promoting the phosphorylation of TBK1 to form a stable tetramer. Phosphorylation of TBK1 exerts its effects by prompting IRF3 and NF-κB to migrate to the nucleus to trigger type I interferon transcription and activate inflammatory pathways

First reported in 2013, cGAS, also known as MB21D1, is predominantly distributed in the cytoplasm, with studies showing it in the cell membrane and nucleus [13]. Structural studies have shown that cGAS cannot function alone in the free state and must be combined with dsDNA [14]. DNA, the most fundamental carrier of life, is restricted to the nucleus and some organelles (e.g., mitochondria). Ectopic DNA is rapidly recognized and subsequently degraded by scavenger cells and extracellular or intracellular ribonucleases. However, tissue damage leads to persistent accumulation of dsDNA as well as exogenous dsDNA invasion, e.g., from pathogens such as viruses, bacteria, transcellular vesicles, or ruptured dying cells, which can be internalized into the cytosol to activate cGAS in various ways [15–17]. Further research has revealed that not all free dsDNA binds to cGAS; only dsDNA longer than 20 bp can combine with cGAS, and dsDNA > 40 bp can form more stable complexes to activate the biological activity of the cGAS protein [18, 19]. dsDNA binds mainly to the surface of the A site of cGAS, whereas the B site binds to another dsDNA as a complementary site. This unique structure facilitates the formation of a 2:2 cGAS:dsDNA bidirectional complex, leading to a conformational change in cGAS and rearrangement of the enzymatic catalytic pocket [20]. Activated cGAS synthesizes the endogenous second messenger cyclic dinucleotide (CDN) cGAMP by catalyzing the synthesis of the substrates guanosine triphosphate (GTP) and adenosine triphosphate (ATP), which then activate STING signaling [21]. Interestingly, STING, whose common names include TMEM173, MITA, and ERIS, is an endoplasmic reticulum (ER) membrane protein that was discovered in 2008 [22, 23]. In its resting state, STING neither directly performs its biological function and nor directly recognizes DNA; instead, STING binds to cGAMP in mammalian cells to activate its biological effects [14]. Upon binding to cGAMP, STING undergoes conformational changes to form a stable tetramer that translocates from the ER to the Golgi apparatus via the ER-Golgi intermediate compartment (ERGIC), recruits TANK-binding kinase 1 (TBK1), and promotes the phosphorylation of TBK1 [24]. Phosphorylated TBK1 is essential for IRF3 and I-kappa B kinase complex (IKK) activity. On the one hand, phosphorylated TBK1 promotes IRF3 phosphorylation, leading to its dimerization and translocation into the nucleus; induces IFN-I expression; and promotes gene expression of inflammatory mediators and chemokines [25]. On the other hand, the IKK-promoted activation of nuclear factor-kappa B (NF-κB), the p50/p65 complex, which acts as a transcription factor translocated into the nucleus, also promotes the expression of inflammatory mediator genes and binds to the IFN-I promoter to aid in its expression [22, 23]. STING binds to cGAMP and translocates from the ER to the Golgi compartment, further activating downstream signaling and regulating multiple biological functions (Fig. 1).

Increasing evidence suggests that the activation of the cGAS-STING signaling pathway is involved in a variety of cellular processes, such as pyroptosis, apoptosis, autophagy, senescence, and IFN-I inflammatory signaling. Furthermore, some research teams have found that activating the cGAS-STING pathway leads to increased IFN-I expression, with a strong, positive association [26]. In contrast, STING knockdown reduces the IFN-I inflammatory signaling pathway activity and inhibits cardiomyocyte apoptosis and inflammatory infiltration in myocardial toxicology experiments [27]. These results suggest that the cGAS-STING signaling pathway plays a key role in the induction of inflammation. Recently, the cGAS-STING signaling pathway was found to be activated in various CVDs to participate in the regulation of immune-inflammatory responses [28], suggesting that it may be a therapeutic target for the treatment of CVDs.

## Overview of CVDs

CVDs such as atherosclerosis (AS) affect tens of thousands of people, are progressively occurring at a younger age, and are triggered by a combination of internal and external influences such as genetic and environmental factors [1, 29, 30]. Several studies have shown that CVDs are associated with age and sex [31, 32]. Specifically, age has been shown to be an independent risk factor for CVDs [33]. Interestingly, men are more susceptible to CVDs, with women developing the disease an average of 10–15 years later [34]. The most common CVDs encountered in clinical practice include atherosclerotic heart disease, congenital heart disease, aortic coarctation/aneurysm, and valvular diseases. Aortic coarctation/aneurysm is the deadliest surgical CVD, with mortality increasing 1–2% per hour after onset and 50% of patients dying outside the hospital [35]. This suggests an urgent need to elucidate the intrinsic mechanisms of CVDs to better prevent and intervene in their development. Substantial evidence confirms that CVD development is related to both innate and adaptive immunity, in which dysregulation of immunity is a major trigger in pathogenesis and for the induction of an acute inflammatory response to tissue injury and infection [36]. Unfortunately, host damage leading to ectopic DNA and invasion by exogenous DNA can cause dysregulation of immune homeostasis, triggering an inflammatory response that can lead to CVDs, making detecting and promptly eliminating the effects of DNA particularly important [37]. Three-prime repair exonuclease 1 (TREX1) is a DNA enzyme that degrades single- and double-stranded DNA. Importantly, TREX1 can degrade DNA in cytoplasmic lysates, thereby preventing accumulation in the cytosol, activating the IFN-I inflammatory response pathway, and protecting the host from inappropriate damage [38]. Morita et al. [39] demonstrated that TREX1 deletion (TREX^−/−^) leads to increased mortality in mice through the IFN-1 pathway, and mice are more likely to develop inflammatory myocarditis, leading to progressive cardiomyopathy and circulatory failure. Similarly, the inability to clear DNA from apoptotic cells in mice lacking macrophage lysosomal DNAase activates an IFN-I-driven inflammatory response [40]. Fortunately, the discovery of a new DNA receptor signal, cGAS-STING, can reverse this phenomenon and potently modulate immune inflammation. Gray et al. [41] found that TREX^−/−^ mice lacking cGAS exhibit complete protection from lethality, have significantly reduced IFN-I-mediated tissue inflammation, and fail to produce autoantibodies. Importantly, in cGAS-deficient (cGAS^−/−^) mice, disrupting IFN-I-dependent signaling results in reduced cardiac expression of inflammatory cytokines and chemokines, decreased cardiac inflammatory cell infiltration, attenuated ventricular dilatation, and improved cardiac function [8]. Therefore, cGAS-STING acts as a DNA receptor signaling pathway to provide novel support for host defense against inflammatory responses resulting from abnormalities in immune homeostasis, thereby providing new targets for treating CVDs.

## Functional role of the cGAS-STING pathway in CVDs

Recent studies have shown that the cGAS-STING pathway activated is closely associated with CVDs, such as AS [42], aortic aneurysms and dissections (AAD) [43], and heart failure (HF) [44] (Table 1). In contrast, this signaling pathway downregulates IFN-I-mediated inflammatory factor release when inhibited, suggesting that it plays an instrumental role in CVD development. Here, we summarize the regulatory role of the cGAS-STING signaling pathway in various CVDs.

**Table 1:** Expression of the cGAS-STING signaling pathway in multiple cardiovascular diseases (CVDs)

Cardiovascular diseases (CVDs)	In vivo	In vitro	cGAS/STING expression	Biological Function	Reference
Atherosclerosis (AS)	HUVECs,VSMCs, macrophages	ApoE^−/−^ mice Ldlr^−/−^ mice (HFD)	+ ^a^, +	Promotes pyroptosis of HUVECs, cell proliferation, phenotypic switching and premature senescence of VSMCs, and foam cell formation and inflammatory responses of macrophages	33,717,842 37,597,406 34,291,051
Aortic aneurysms and dissection(AAD)	VSMCs	STING^−/−^ mice (Angiotensin II)	/^b^, +	STING^−/−^ mice inhibits DNA damage response, inflammatory response, dedifferentiation, and cell death in VSMCs	31,887,080 37,555,319
Heart failure (HF)	NRCMs	C57BL/6 J mice (TAC or AB band)	+ , +	Promotes myocardial hypertrophy, fibrosis, apoptosis, autophagy, macrophage infiltration, and ventricular remodeling	321,063,79 32,383,996
Myocardial infarction (MI)	Macrophages	cGAS^−/−^ mice STING^−/−^ mice (Ligation of the LCA or LAD)	+ , +	cGAS^−/−^ mice increases survival and heart function, attenuates cardiac remodeling, and promotes macrophage-to-repair cell conversion	29,106,401 29,437,120
Heart transplantation(HT)	HL-1 cells	cGAS^–/–^ mice BALB/C mice	+ , +	cGAS^−/−^ mouse donors prolonged graft survival and decreased levels of proinflammatory cytokines TNF-α, IL-18, and IL-6	37,061,907
Viral myocarditis(VMC)	THP-1 cells	Trim18^−/−^ mice (RNA virus CVB3)	/, +	Inhibition of TBK1 phosphorylation fails to interact with STING and suppresses viral invasion	35,909,127
Diabetic cardiomyopathy (DCM)	H9C2 cells NMCMs	Db/db mice (HFD) C57BL/6 J mice(STZ + HFD)	+ , +	Promotes pyroptosis of H9C2	35,538,059 35,235,096
Hypertension	BV2 cells	cGAS^−/−^ mice (Angiotensin II)	+ , +	Promotes neuroinflammation sympathetic overactivation induces hypertensive myocardial injury	36,070,120

HUVEC, Human umbilical vein endothelial cells; VSMC, Vascular smooth muscle cells; NRCMs, Neonatal rat cardiomyocytes; NMCMs, Neonatal mouse cardiomyocytes; LCA, Left coronary artery; LAD, Left Anterior descending artery; TAC, Transverse aortic constriction

^a^ “ + ”: Increased expression. ^b^ “/”: There was no indication of expression in the reference

### AS

Inflammatory infiltration, lipid deposition, and foam cell formation in atherosclerosis (AS) predominantly affect the medium- and large-sized arteries, causing luminal narrowing and tissue ischemia and resulting in severe clinical symptoms and complications with a poor prognosis. AS is currently the main killer and the incidence is increasing worldwide. Experimental data suggest that IFN-I is involved in the entire process of atherogenesis, affecting macrophages to enhance phagocytosis and the formation of foam cells and extracellular traps; in addition, IFN-1 can change the phenotype of dendritic, T cells, and B cells, causing immune responses that accelerate further AS deterioration [45]. Recent studies have shown that the cGAS-STING signaling pathway involves immunoinflammatory recognition signals that senses ectopic DNA to activate IFN-I, which provides a new vision for understanding the pathogenesis and continued progression of AS [20, 46]. Wan et al. [42] found that cGAS-STING regulates phenotypic switching, proliferation, and apoptosis in vascular smooth muscle (VSMC), which can be directly activated by intracellular Ca^2+^ (i.e., an inflammatory activator) concentrations to promote AS plaque rupture by activating STING expression. Similarly, Bi et al. [47] found that cGAS or STING deficiency blocks plaque vulnerability by inhibiting the IFN-I pathway-induced premature senescence and phenotypic transition of VSMC, attenuating VSMC fibrous cap loss and thinning. The rupture of AS plaques seriously jeopardizes health and causes irreversible organic damage. Therefore, inhibiting the cGAS-STING signaling pathway may be a potential target for the treatment of AS.

### AAD

AAD are aggressive and one of the most difficult surgically treated conditions for cardiac and macrovascular surgeons. In the United States, a study of residents of Olmsted County, Minnesota found that the incidence of aortic lesions was approximately 7.7/1,000,000 [48]. In addition, the incidence of AAD is twice as high in men as in women and increases with age [49]. Furthermore, the disease onset is accompanied by a variety of clinical manifestations such as coma, crushing pain in the anterior thoracic region, elevated blood pressure, pericardial tamponade, and renal failure, resulting in poor prognosis. The mechanism of AAD development is predominantly progressive smooth muscle cell (SMC) loss and extracellular matrix (ECM) degradation and depletion, leading to aortic aneurysm, dissection, and rupture; however, the underlying mechanisms are still being explored [50]. Recent studies have revealed the role of the cGAS-STING signaling pathway in the pathogenesis of AAD [43, 51]. Specifically, Luo et al. [43] showed that increased cytoplasmic DNA release from SMCs and macrophages in AAD patient tissues activates STING. DNA from damaged SMCs in sporadic AAD induced by a high-fat diet and angiotensin (Ang) in mice is phagocytosed by macrophages, activating STING and its downstream IRF3, which directly induces matrix metalloproteinase-9 expression and ECM degeneration, leading to aortic thickening, dissection, and rupture. Compared with wild-type mice, this phenomenon is inhibited in STING-deficient mice (STING^gt/gt^). In addition, reactive oxygen species (ROS)-mediated DNA damage results in the progressive release of DNA fragments, which subsequently stimulate the cGAS-TING-TBK1-IRF3 signaling pathway and promote aortic SMC death [46]. In a single-cell study, STING-IRF3 signaling induced chromatin remodeling that drove SMC from a contractile to an inflammatory phenotype; however, in STING^−/−^ mice, the aortic stress-induced shift of SMC to an inflammatory phenotype was blocked, and the SMC population was preserved [51]. Therefore, the cGAS-STING signaling pathway is a new target class for future AAD therapies.

### HF

HF is characterized by systolic or diastolic dysfunction of the heart, insufficient peripheral ejection, and inability to supply enough oxygen to meet the metabolic requirements of the entire organism, resulting in clinical symptoms [52]. The main clinical manifestations are edema of the lower extremities, dyspnea, pink foamy expectorations, pulmonary edema, pleural effusion, and progressive exertional failure that interferes with daily life [53]. HF has become an epidemic in the modern world, affecting approximately 1–2% of adults [54]. In Europe and North America, the lifetime risk of developing HF is approximately one in five for people over 40 years of age [55]. A growing body of research supports the idea that myocardial hypertrophy and the activation of cardiac inflammation, in which cGAS-STNG plays an important role, are key triggers of HF in humans and animals [56, 57]. In a study by Hu et al.[9], pathological myocardial remodeling and ventricular dysfunction occurred in transverse aortic constriction (TAC) treatment-induced HF, where cGAS-STING signaling was activated. Interestingly, when the upstream expression of cGAS was decreased, the survival of mice after TAC improved, with preserved myocardial contractility and attenuated myocardial hypertrophy, fibrosis, and pyroptosis. Similarly, Zhang et al. [44] found that STING-deficient mice undergoing aortic banding (AB) surgery have attenuated AB-induced cardiac hypertrophy and an inhibited macrophage infiltration and IFN-I-mediated inflammatory response. Interestingly, in another study of AB surgery, STING overexpression was shown to improve cardiac function and significantly attenuate cardiac hypertrophy, fibrosis, and inflammation; the in vitro STING overexpression in Ang II-induced cardiomyocytes significantly suppressed the cardiomyocyte cross-sectional area and atrial natriuretic peptide (ANP) mRNA levels [57]. Thus, the cGAS-STING signaling pathway clearly plays an important role in HF. Although the exact mechanism has remained controversial, the pathway may still be targeted for the treatment of other CVDs; further clarification may be needed to develop small-molecule inhibitors for applications in HF.

### MI

MI, a disease that develops in the elderly, has a sudden onset, high lethality, and high disability [58]. Once the attack intensifies to persistent, severe retrosternal pain, patients experience fear, anxiety, and other disturbing emotions, especially a near-death feeling, resulting in lasting psychological trauma [59]. The interruption of blood flow in the area of MI can simultaneously lead to massive cardiac cell death, releasing a large number of molecules associated with the pattern of injury and causing an inflammatory response and infiltration of inflammatory cells [60]. Phagocytosis of injured cardiac cells by inflammatory cells and some matrix debris activates repair pathways to form a fibrotic scar and maintain cardiac integrity but sclerotizes the ventricular wall to further compromise cardiac function [61]. Complex mechanisms exist during this period, and prolonged activation of inflammatory signaling and infiltration of inflammatory cells exacerbate adverse remodeling and concomitant injuries. Recent studies have shown that cGAS-STING is involved in inflammatory activation pathways after MI. King et al. [8] found that the phagocytosis of necrotic cells and cellular debris by macrophages after MI promotes the lethal process of MI by activating the STING-IRF3 mutation. I-IFN induction is inhibited in cGAS^−/−^, STING^gt/gt^, and IRF3^−/−^ mice, with STING^gt/gt^ and IRF3^−/−^ mice showing the same expression patterns and survival rates of 82% (39/47), 77% (24/31), and 98% (44/45), respectively. Cao et al. [62] found that the loss of cGAS function eliminates the induction of key inflammatory programs, such as inducible nitric oxide synthase (iNOS), and promotes macrophage conversion to a reparative phenotype, thereby enhancing repair and improving hemodynamic performance. Thus, inhibiting the cGAS-STING pathway could be a new pharmacological target for the treatment of MI.

### Cardiomyopathy

Cardiomyopathy, a relatively rare cardiac disease with multiple causes, can be divided into primary cardiomyopathies, which include hereditary, mixed, and acquired types, and secondary cardiomyopathies, which include dilated, hypertrophic, and restrictive types [63]. However, with a poor prognosis and limited therapeutic options, cardiomyopathy has been reported in nearly 50% of children and adolescents who die suddenly or undergo heart transplantation [64]. The pathogenesis of cardiomyopathies remains unclear. The most widely studied is hypertrophic cardiomyopathy, for which there is now extensive evidence of the involvement of cGAS-STING in the development. For example, in a mouse model of diabetic cardiomyopathy, Yan et al. [10] and Ma et al. [65] found increased levels of ROS in the myocardial tissue by dihydroethidium (DHE) staining, an increase in ectopic DNA by co-localization of dsDNA with mitochondrial somatic tracking, and an increase in cardiomyocyte death by dUTP nick end labeling (TUNEL) staining. Mechanistic studies revealed that it was associated with the activation of the cGAS-STING-induced cardiomyocyte focal death, worsening the progression of diabetic cardiomyopathy and leading to myocardial hypertrophy. Myocardial hypertrophy is closely associated with certain types of cardiomyopathy and is a major marker in patients with chronic kidney disease (CKD). Han et al. [66] developed a CKD model using cGAS-deficient (cGAS^−/−^) or STING-deficient (STING^−/−^) mice and were able to inhibit cardiac hypertrophy through the NF-KB pathway.

As previously mentioned, cGAS-STING is closely related to CVDs, and targeting this pathway may be a new means of treating CVDs. In addition, once CVDs occur, they impose a huge economic and psychological burden on patients and their families; the best way to address this is through primary prevention, understanding the risk factors that may activate the pathway daily, and early-stage prevention.

## Risk factors for activating cGAS-STING in daily life

**Fig. 2:**
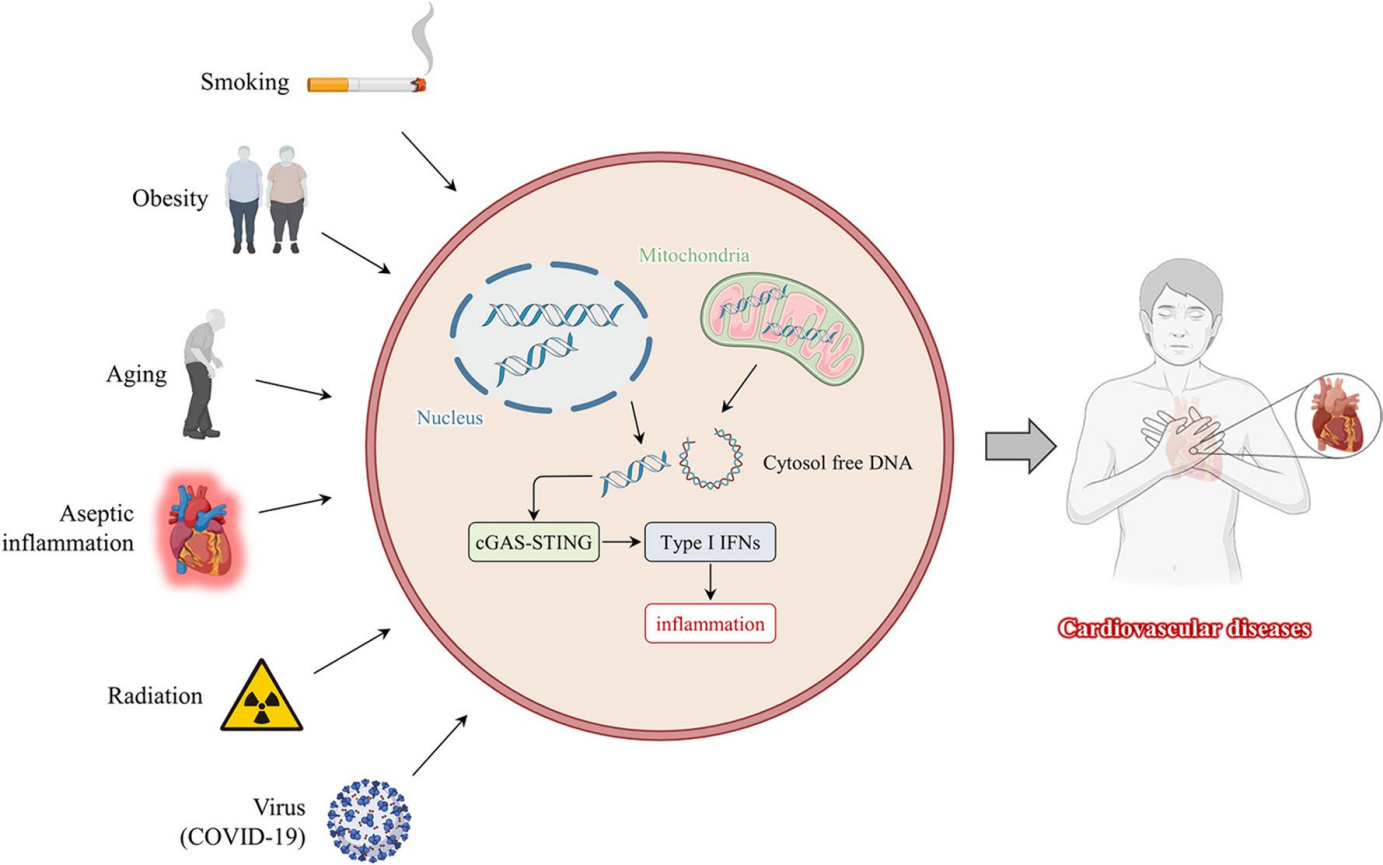
Risk factors for activating cGAS-STING in daily life. Smoking, obesity, aging, radiation, aseptic inflammation, and viruses (COVID-19) contribute to organismal damage. DNA from mitochondria or nucleus is released into the cytoplasm, activating the cGAS-STING signaling pathway and type I interferon transcription, causing an excessive inflammatory response resulting in cardiovascular diseases and poor prognosis

Disease complexity and heterogeneity are closely related to genetic, lifestyle, and environmental factors [67]. Although genetics are critical to disease development, some studies have shown that lifestyle and environmental factors can alter genetic behavior [68]. CVD triggers include risk factors such as smoking [69], obesity [70], and radiation [71] (Fig. 2). Understanding the role that lifestyle and environmental factors play in activating the cGAS-STING pathway is critical and provides a basis for guiding us to live healthier lives and avoid exposure.

### Smoking

Although smoking kills approximately 6 million people annually, the deaths are preventable [72]. Thousands of chemicals in cigarette smoke contribute to the development of CVDs through a variety of mechanisms such as inflammation, dysfunctional hemostasis, and endothelial dysfunction [73]. Smoking is a leading cause of CVDs, with 140,000 premature deaths from CVDs each year [72]. The smoking population is relatively small in developed countries. Of note, the smoking population in Japan is 29.4% male and 7.2% female, and unfortunately, the average smoking prevalence rate of middle-aged males remains approximately 40% [74]. Smoking is an important underlying trigger for CVD development, and cardiovascular vigilance will need to be a permanent concern in the coming decades. Ueda et al. [75] found increased levels of free DNA in the serum of AS patients in a smoking population, and overexpression of free DNA was also detected in AS plaques, which activated the cGAS-STING pathway and became a AS marker. Clinical and experimental data suggest that cigarette smoking and secondhand smoke are equally detrimental in CVDs such as coronary artery disease, hypertension, and cardiac systolic and diastolic abnormalities. Liu et al. [69] found that exposing wild-type mice to sidestream cigarette smoke caused mtDNA release and activation of cGAS-STING signaling induced myocardial autophagy to disrupt cardiac homeostasis, and this effect removing in Beclin1 haplotype-sufficient (Beclin1^±^) mice. Long-term chronic obstructive pulmonary disease (COPD) is one of the most important causes of cardiac impairment and failure. In COPD induced by exposing mice to high levels of cigarette smoke, mitochondrial DNA (mtDNA) release from respiratory tract injury activates the cGAS-STING-IRF3 pathway to cause an excessive inflammatory response and exacerbate lung injury; however, the pathway is inhibited in cGAS-deficient (cGAS^−/−^) and STING-deficient (STING^−/−^) mice [76]. Therefore, smoking cessation is an urgent issue for patients with CVDs, and avoiding secondhand smoke to reduce exposure may lead to a better prognosis for patients with CVDs.

### Obesity and aging

People with a body mass index (BMI) > 25.0 kg/m^2^ are classified as obese [77]. During the last decades of the twentieth century, changes in the dietary structure of the population have resulted in a shift from having a standard weight or being underweight to being overweight or obese. Each 1 kg/m^2^ increase in BMI in obese patients increases the risk of HF and coronary heart disease by 12% and 7%, respectively, and a BMI reduction to that of the 1980s is needed to mitigate the ongoing weight damage [78, 79]. The mechanisms through which obesity affects CVDs are currently being investigated. Recently, cGAS-STING has been shown to play a role in high-fat-induced myocardial injury in mice [80]. For example, Mao et al. [70] found that metabolic stress in obese patients induces endothelial cell inflammation, and the cGAS-STING pathway is active in PA-induced aortic endothelium, whereas in vivo experiments have shown that STING-deficient (STING^gt/gt^) mice prevent high-fat diet-induced vascular inflammation, insulin resistance, and glucose intolerance. Similarly, Gong et al. [81] found that cGAS-STING is activated in mice with high-fat diet-induced cardiac abnormalities, leading to impaired myocardial contractile function. However, aging is commonly accompanied by metabolic disorders (e.g., obesity), which induce chronic inflammation and lead to cardiomyocyte hypertrophy, fibrosis, and death, thereby increasing the CVD risk [82]. Takahashi et al. [83] found that when DNA enzymes (i.e., DNase2 and TREX1) are progressively downregulated in senescent cells, the aberrant accumulation of cytosolic DNA activates cGAS-STING, which induces senescence-associated secretory phenotypes (SASPs) via IFN-β. Notably, mouse senescent myocardium is triggered by the inflammatory cytokines interleukin (IL)-1β, IL-6, and IL-18 to release mtDNA, which activates cGAS-STING to induce SASP development [84]. Hu et al. [85] found that immunohistochemical staining of aortic tissue sections from aging patients revealed increased expression of cGAS, STING, and p-IRF3 relative to that in younger patients; the in vitro inhibition of cGAS attenuates the aortic endothelial senescence markers p53, p21, and β-galactosidase, demonstrating the potential value in the prevention of CVDs in the elderly. Thus, obesity and aging contribute to activating the cGAS-STING pathway. Obesity can be controlled through healthy means such as exercise and reduced food intake, while normal aging is a natural process that is not overly concerning.

### Aseptic inflammation

Aseptic inflammation is the absence of microbial infection and features of intractable chronic inflammation that promote the development of autoimmune, metabolic, neurodegenerative, and cardiovascular diseases [86]. Inflammation requires either exogenous (e.g., microorganisms, bacteria, and viruses) or endogenous (e.g., autologous DNA, proteins, and lipids) ligands that activate downstream inflammatory signals [87]. The sources of inflammatory cytokine production in CVDs include almost all cardiac cells such as endothelial cells, cardiomyocytes, and resident macrophages [88–90]. Recent reports have suggested that the cGAS-STING signaling pathway plays an important role in cardiovascular inflammation [91]. The PA-induced release of mtDNA from endothelial cells activates the cGAS-STING-IRF3 signaling pathway to promote increased levels of inflammatory cytokines IL-1β, IL-6, and NLRP3 inflammasome, leading to pyroptosis onset and affecting the formation of AS; using small interfering RNAs cGAS or STING inhibits this process [92]. Guo et al. [56] found that iNOS deficiency inhibits mtDNA release to attenuate stress-induced aseptic cardiac inflammation; however, activated cGAS-STING attenuates its cardioprotective function, suggesting that preventing sterile inflammation is particularly critical for CVDs. However, sterile inflammation can be incomprehensible in the daily lives of the less cognizant general population, which is a major challenge. Better popularization of scientific materials and easy-to-understand language is required to publicize the dangers of aseptic inflammation, avoid overexercising to increase cardiorespiratory injury, control blood pressure, and reduce heat, acidity, and other factors that may cause inflammatory lesions.

### Radiation

With the development of medical technology, radiation therapy and diagnosis have become essential medical support tools for cancer patients [93]. Different cancer types may require different radiation therapy doses, and clinically significant CVDs may occur when radiation therapy doses exceed 30 Gy [94]. The breast cancer radiation treatment group has been shown to have a 27% increase in mortality from heart disease compared to that in the non-radiation group, and a case–control study of Hodgkin's lymphoma survivors found an increased risk of coronary heart disease of 7.4% per Gy [95, 96]. Radiation therapy induces cardiovascular ROS, and chronic inflammation leads to cardiac remodeling, including fibrosis, apoptosis, and hypertrophy. Elevated inflammatory markers and enhanced immune response modulation have been found in cancer survivors [97]. Philipp et al.[71] observed the in vitro radiation of human coronary artery endothelial cells; proteomics revealed that few protein changes are induced by low (0.25 Gy) and medium (0.5 Gy) doses of radiation, but DNA damage, increased ROS levels, and inflammatory reactions occur after high doses of radiation (2 and 10 Gy). Interestingly, the protein expression in the cGAS-STING pathway has been associated with an increased radiation dose, with a strong effect at 10 Gy after one week. Pain in patients with cancer seriously affects their psychological and physical well-being, and paying attention to the radiation dose during treatment and diagnosis is even more important to avoid complications due to improper activation of the cGAS-STING pathway, which further damages the cardiovascular system.

### Viral (i.e., severe acute respiratory syndrome coronavirus 2 [SARS-CoV-2])

Both RNA and DNA viruses are strongly associated with CVDs; however, the ability of RNA viruses to rapidly mutate and recombine is more dangerous [98]. Unfortunately, the December 2019 outbreak in Wuhan was caused by a novel coronavirus that became a global epidemic and was named coronavirus disease 2019 (COVID-19) [99]. As research progressed, COVID-19, identified as a type B coronavirus that was closely related to SARS-CoV and a bat coronavirus, was renamed SARS-CoV-2 [100]. Patients with SARS-CoV-2 pneumonia presenting with cardiovascular complications have a variety of clinical manifestations, with initial symptoms of palpitations and chest tightness [101]. SARS-CoV-2 causes myocardial injury, arrhythmia, and HF, with incidences as high as 19.7%, 16.7%, and 23%, respectively [102]. Cardiovascular complications due to SARS-CoV-2 are associated with systemic inflammatory storms, oxidative stress, and DNA damage, and the cGAS-STING pathway can activate these factors [103, 104]. Mechanistically, Elahi et al. [105] also described that, in theory, the cGAS-STING signaling pathway leads to SARS-CoV-2-induced endothelial dysfunction and coagulation disorders, which may require more experimental evidence. Fang et al. [106] found that the aberrant activation of cGAS-STING signaling in viral myocarditis leads to an inflammatory infiltrate of I-IFN transcription that damages the myocardium, which is inhibited in T-cell receptor interacting molecule (TRIM)-deficient mice. Although SARS-CoV-2 is still present and constantly mutating, it does not cause anxiety or panic. Now that vaccines and drugs are available, the virus has become less pathogenic and has a symbiotic relationship. However, older populations and those with underlying medical conditions such as high blood pressure, diabetes, and immune deficiencies still need to take precautions.

## Therapeutic targets of the cGAS-STING pathway in CVDs

As described previously, the cGAS-STING signaling pathway is involved in the development of multiple CVDs and risk factors to avoid overexposure. Notably, therapeutic agents are needed for patients with CVDs in order to target the pathway to block the inflammatory cascade response, especially I-IFN transcription, which is difficult and requires a long-term commitment. In a phase II clinical trial of squamous cell carcinoma of the head and neck, the cGAS inhibitor MK-1454 was well tolerated clinically, providing great confidence in its clinical applications (clinicaltrials.gov: NCT04220866). Therefore, small-molecule inhibitors targeting the cGAS-STING signaling pathway in CVDs are promising. In this section, we summarize the most clinically promising small-molecule inhibitors of cGAS and STING that are used in cardiovascular research (Tables 2 and 3).

**Table 2:** cGAS inhibitors in cardiovascular disease (CVDs)

Categories	Compound name	Structure	Pharmacological mechanisms	Reference
RU series compounds	RU.521		Competes with ATP and GTP for the active site of cGAS, decreasing cGAMP synthesis	28,963,528
	RU.365			
Antimalarial drugs	Quinine		Barring activation of cGAS by dsDNA through embedding, active-slot binding, and covalent binding interactions	27,813,878
	Quinacrine			
	Chloroquine			
	Hydroxychloroquine			
	Primaquine			
	X6			
	9-Amino-6-chloro-2-methxyacridine			
Suramin			Direct replacement of dsDAN in cGAS prevents cGAS activation from mediating I-IFN transcription	33,833,439
Aspirin			Maintains the acetylated inactive state of cGAS on Lys384, Lys394, or Lys414 and avoids attack by dsDNA	30,799,039
PF Series Compounds	PF-06928215		Interaction of Lys362 with Lys350 of cGAS, inhibiting the catalytic activity of ATP and GTP on cGAS interferes with the production of cGAMP	28,934,246 32,512,189

**Table 3:** STING inhibitors in cardiovascular disease (CVDs)

Categories	Compound name	Structure	Pharmacological mechanisms	Reference
Nitrofuran derivatives	C-170		Palmitoylating cys99 on the structure of STING proteins, alters the molecular characteristics	37,138,486
	C-171			
	C-176			
	C-178			
3-acylaminoindole derivative	H-151		Palmitoylating cys99 on the structure of STING proteins, alters the molecular characteristics	35,127,372
Astin C			Specifically binds to the C-terminal activation pocket of STING, displaces CDN, and prevents IRF3 recruitment and activation	30,566,866
Tetradroisoquinolone acetic acid	Compound 18		Occupies a large symmetric binding pocket in STING proteins and can displace cGAMP and block all STING signaling	30,655,953
Nitro-fatty acids derivatives	NO_2_-cLA		Nitroalkylation of the sulfhydryl groups of cys99 and cys88 located in the N-terminal region of STING blocks palmitoylation	30,061,387
	NO_2_-OA			

### cGAS inhibitors

Based on the molecular structure and DNA recognition of cGAS, several compounds have been developed that inhibit the catalytic activity of cGAS or compete with cGAS for DNA binding. Briefly, cGAS is not sensed when small-molecule inhibitors that are unable to bind to ATP and GTP substrates or their product (i.e., cGAMP) compete for the cGAS active site and lose catalytic activity or when DNA competes with other small molecules for binding [107, 108]. Representative compounds include the RU series compounds [109], antimalarials [110], and PF Series Compounds [81].

#### RU series compounds

The RU family of small-molecule compounds includes RU.521 and RU.365. The most studied cardiovascular RU compound is RU.521, possibly because of its minimal toxicity at ≥ 50% loss of cell viability at IC50; RU.521 competes with ATP and GTP for the active site of cGAS [111]. Wu et al. [112] found a significant prolongation of the graft time in mouse hearts using cGAS^−/−^ mouse heart donors, and the same results were obtained by intraperitoneal injection of 10 mg/kg/day RU.521 (dimethyl sulfoxide using corn oil dilution) in recipient mice, starting 1 d before surgery. The results are related to the reduction of inflammatory factors IL-6, TNF-α, and IFN-γ and leukocyte infiltration. Yu et al. [85] found that in six-month-old mice, continuous administration of RU.521 for six months inhibits cGAS expression and I-IFN transcription to reduce aortic endothelial cell senescence. Interestingly, a study linking neuroinflammation in the paraventricular nucleus of mice with hypertensive heart disease found that an intra-feeding perfusion of RU.521 blocked microglia autophagic fluxes to attenuate the hypertensive myocardial injury caused by neuroinflammation and sympathetic overactivation [113]. Meanwhile, RU.521 plays a critical role in attenuating sepsis-induced cardiac dysfunction [109], endothelial focal death-induced AS [92], ischemia–reperfusion-induced myocardial apoptosis, and cardiac dysfunction [114]. These findings provide a new phase in the clinical application of RU.521 for the treatment of CVDs.

#### New targets of antimalarial drugs

Until quinine (QN), a valuable synthetic antimalarial drug, was synthesized and purified during World War II, malaria was treated empirically using the bark of the cinchona tree, a Latin American tropical plant [115]. Recent studies have shown that some classical antimalarial drugs such as quinoline (i.e., chloroquine [CQ] and hydroxychloroquine [HCQ]) and acridine (QC) can treat several inflammatory diseases [116, 117]. Importantly, they block the triggering of cGAS activity using dsDNA via embedding, active groove binding, and covalent binding interactions [118]. In a study of ultraviolet (UV)-induced acute inflammation, B6 mice were continuously injected with HCQ (25 mg/kg/day) for three weeks. The I-IFN expression decreased in the skin and blood after six hours of UV irradiation, and the early IFN-I response in the skin and infiltration of inflammatory cells were dependent on the presence of cGAS [119]. Interestingly, An et al. [120] found that oral administration of X6 or HCQ (25 mg/kg/day) in TREX1-deficient mice from birth was able to treat cGAS-STING pathway active-mediated autoimmune myocarditis attenuating endomyocardial fibrosis and inflammation, and compared to HCQ, X6 significantly reduced cGAMP expression in the heart in an AGS model, with a therapeutic effect of better. These studies suggest that antimalarial drugs have new clinical applications and are significant for treating the cGAS-STING signaling pathway, a pharmacological CVD target.

#### PF series compounds

Hall et al. [121] used a high-throughput drug screening technique to identify PF-06928215 among the PF series of compounds. Using a novel fluorescence polarization assay, they also found that PF-06928215 interacted with Lys362 and Lys350 in cGAS, inhibited the catalytic activity of cGAS by ATP and GTP, and interfered with the production of cGAMP. Notably, their study also showed that PF-06928215 inhibited the activity of cGAS using as little as 200 nmmol/L, which demonstrated a high affinity. In studies investigating the effects of PM2.5 on senescence of lung cells, Wang et al. [122] showed that PF-06928215 was able to inhibit lung epithelial cell senescence induced by a cGAS-STING signaling pathway-mediated release of inflammatory factors IL-6, IL-8, and TNF-α. In a mechanistic study of high-fat diet-induced cardiac remodeling and contractile dysfunction, Gong et al. [81] discovered that PF-06928215 inhibited cGAS activity, prolonged palmitic acid-induced peak contraction and maximal shortening velocities of cardiomyocytes (+ dL/dt), and treated myocardial contractile dysfunction without eliciting any mechanical response on its own. Treatment effects of PF-06928215 on cardiovascular disease through inhibition of the cGAS-STING signaling pathway have not been studied in vivo. However, indirect evidence provides an essential research base for in vivo applied therapy. Further research with PF-06928215 may make it possible to treat cardiovascular diseases through inhibition of the cGAS-STING signaling pathway.

### STING inhibitors

STING, an intermediate pathway protein, has been associated with several CVDs. Competitive antagonists have been designed based on molecules occupying CDN binding sites according to molecular crystal structure dynamics or palmitoylation of the STING protein structural domains Cys88 or Cys91 that alter molecular properties.

#### Nitrofuran derivatives

Nitrofuran derivatives include C-176, C-171, C-178, and C-170 [20]. C-176 has become the most studied compound in cardiovascular research. Nitrofuran derivatives can palmitoylate Cys99 on STING proteins to alter their molecular properties and affect their response-mediated I-IFN transcription [123]. Hua et al.[124] showed that in α-MyHC-induced autoimmune myocarditis (EAM), consecutive intraperitoneal injection of 1 μmol of C-176 for 14 days results in an inflammatory response in EAM, with IFN-β, TNF-α, CCL2, and F4/80 expression that is ameliorated by blocking macrophage STING expression. In an in vitro study, C-176 blocked the PA-induced NLRP3-mediated endothelial cell pyroptosis and I-IFN transcription. Pham et al.[125] identified that consecutive 1 μmol intraperitoneal injections of C-176, three times a week for 12 weeks in an AS model inhibits STING-associated vascular inflammation and macrophage activation to attenuate AS formation. Importantly, C-176 also has a therapeutic role in MI [126], diabetic cardiomyopathy [10], and plaque stability [47]. In addition, the 3-acylaminoindole derivative H-151 has the same target as the nitrofuran derivative. In a mouse model of myocardial ischemia–reperfusion injury using preoperative intraperitoneal injection of 750 nmol of H-151, blocking STING attenuates the I-IFN-mediated inflammatory response and macrophage infiltration, increases the left heart ejection fraction, and reduces the extent of infarcts to maintain cardiac function [11, 127]. Therefore, developing pharmacological targets against STING for CVD treatment is essential.

#### Astin C

Li et al. [128] isolated and purified the cyclic peptide Astin C from the Chinese herb, Aster. In subsequent studies, they also found that, owing to the specificity of its molecular crystal structure, Astin C specifically binds to the C-terminal activation pocket of STING and inhibits inflammatory signaling by preventing the recruitment and activation of IRF3 on STING signalosomes through CDN displacement. Furthermore, mice injected intravenously with Astin C compound formulations (2 or 4 mg/kg) have a 100% survival rate within seven days, and decreased levels of the pro-inflammatory factor, IFN-β, as detected by serum enzyme-linked immunosorbent assay (ELISA) assay after treatment [128]. Significantly, Astin C attenuates in vitro myocardial contractile dysfunction in palmitate-induced cardiomyocytes by inhibiting the cGAS-STING signaling pathway in experiments of high-fat diet-induced cardiac remodeling and contractile abnormalities [81]. This in vivo and in vitro evidence of the safety and applicability of Astin C provides confidence and suggests that with further research, the use of Astin C as a pharmacological target for STING in the treatment of CVDs may become a reality.

## Future expectations

Continued basic and clinical research has demonstrated that the cGAS-STING signaling pathway plays a vital role in CVDs. Recently, this pathway has been shown to be activated in MI, AS, and HF, affecting I-IFN transcription to promote an inflammatory cascade response. These findings suggest that the cGAS-STING pathway is of broad interest in the cardiovascular field, including risk factor investigation and the development of pharmacological targets against its molecular crystallographic properties. As a result, we anticipate future cardiovascular clinical applications of cGAS-STING based on these characteristics, new technological features, and achievements.

In terms of the current therapeutic developments in CVDs, no optimal method to eradicate lesions exists. Diseases that do not require surgical intervention include mild or moderate stenosis of the coronary artery, AS, hypertension, and early HF that requires continuous drug therapy. However, pharmacological interventions are not specific and may strain other organs. Surgical treatments are also necessary, including those for severe congenital heart disease, heart transplants, aortic dissection, and genetically related Marfan syndrome. Surgery eradicates these lesions, but with physical and financial burdens, as well as the possibility that the most severe complications can lead to loss of life. This highlights the urgent need for more precise therapies to intervene effectively and promptly in CVDs to prevent deterioration and bodily damage. Sequencing technologies are evolving rapidly. Traditional sequencing microarrays and RNA sequencing, for example, involve averaging gene expression differences in different cell populations in heterogeneous populations reported as a single data point, which is insufficiently precise. This is avoided by single-cell sequencing, which uses fluorescence-activated or conjugated magnetic bead-assisted methods to categorize target cell populations and analyze them individually [129]. In a mouse aortic aneurysm model, single-cell sequencing revealed that increased STING expression in smooth muscle and macrophage subpopulations led to smooth muscle death and dsDNA release, which activated the macrophage STING-TBK1-IRF3 pathway, leading to increased expression of matrix metalloproteinases (MMPs), which degrade the ECM. However, this phenomenon can be inhibited by the STING pathway inhibitor amlexanox [43]. Clustered regularly interspaced short palindromic repeats (CRISPR)-CRISPR-associated nuclease 9 (Cas9), a simple and easy-to-operate gene editing technology that has a wide range of applications in targeting and preventing severe diseases, including CVDs, has been one of the greatest advances in biomedicine in the last two decades [130]. Zhao et al. [131] revealed that CRISPR-Cas9 gene editing delivered by an adeno-associated virus ameliorates familial hypercholesterolemia due to low-density lipoprotein receptor (LDLR) mutations, reduces the aortic AS plaque area, and attenuates inflammatory infiltration. Human papillomavirus (HPV) infection is closely associated with CVD development [132], but the underlying mechanisms are unknown. Using CRISPR-Cas9, Gusho et al. [133] discovered that HPV-infected cells can block STING and IRF3 activation after cGAS knockdown. These results illustrate that single-cell sequencing and CRISPR-Cas9 technology can be applied for the development of pharmacological targets for the cGAS-STING signaling pathway, making precise treatment of CVDs possible.

Neutrophils are one of the most common effector cells of the innate immune system and play an instrumental role in cardiovascular pathophysiology, in addition to their anti-infective function [134, 135]. Neutrophil extracellular traps (NETs) released by activated neutrophils play a crucial role in inflammatory injury [136]. Numerous recent studies have shown that NETs are associated with CVDs, such as AS [137], myocarditis [138], and MI [139], which may be related to the unique cell death program of neutrophils called "NETosis,” “NETosis” releases granules, cytoplasmic guanosine-conjugated chromatin, and bactericidal proteins to form a reticulum during cell death that plays a crucial role in host defense, autoimmunity, and blood coagulation [140]. The "NETosis" structure also includes many neutrophil derivatives such as dsDNA, myeloperoxidase (MPO)-binding DNA, citrullinated histones, and neutrophil elastase, which lead to ROS bursts that mediate tissue damage [141]. Importantly, the neutrophil derivatives in NETs are associated with clinical variables; in 2013, Borissoff et al. [142] showed a positive correlation between the degree of coronary AS by imaging NETs markers. Kang et al. [143] demonstrated that NETs can activate the STING pathway, and inhibiting the I-IFN response enhances neovascularization and vascular repair. However, the cGAS-STING signaling pathway acts as a DNA receptor that senses cytoplasmic DNA to mediate I-IFN production. Based on the above results, we speculate that the cGAS-STING signaling pathway can mediate the function of NETs to influence CVD development, providing a new direction for future therapy.

In summary, an in-depth study of cGAS-STING in CVDs can combine basic and clinical research. New technologies and cutting-edge developments can be combined, which may provide completely new therapeutic approaches to serve patients with CVDs in the future to alleviate the suffering caused by the disease.

## Conclusion

The cGAS-STING signaling pathway is closely related to CVD development. This primer enables readers to better understand the role of cGAS-STING in CVDs, not through over-exploration of the mechanism but rather by serving as a tool to better focus on and develop health, avoid exposure, and learn about the emergence of new therapeutic cardiovascular targets. Recently developed small-molecule inhibitors targeting this pathway have achieved some success in animal experiments. We expect more investigators to study this pathway, including in fields other than CVDs. Simultaneously, we intend to conduct additional, faster clinical trials involving these small-molecule inhibitors or their derivatives to obtain more evidence and provide better clinical treatment for patients.

## Data Availability

No new data sets were used for analysis in this review.

## Abbreviations

CVDs: Cardiovascular diseases
cGAS: Cyclic guanosine monophosphate-adenosine monophosphate synthase
STING: Stimulator of interferon gene
IFN-I: Type I interferon
NLRP3: Nucleotide-binding oligomerization domain-like receptor pyrin domain containing 3
ER: Endoplasmic reticulum
TBK1: TANK-binding kinase 1
AS: Atherosclerosis
TREX1: Three-prime repair exonuclease 1
AAD: Aortic aneurysms and dissection
HF: Heart failure
TAC: Transverse aortic constriction
MI: Myocardial infarction
COPD: Chronic obstructive pulmonary disease
AIM2: Absent in melanoma 2
LDLR: Low-density lipoprotein receptor
NETs: Neutrophil extracellular traps
